# Case Report: Post-LASIK exacerbation of granular corneal dystrophy type 2: a familial case with *TGFBI* mutation

**DOI:** 10.3389/fmed.2025.1721130

**Published:** 2025-12-09

**Authors:** Longhao Kuang, Xuanjun Zhang, Yufang Wang, Qing Zhan, Jingjing Su, Baotao Lin, Ping Guo

**Affiliations:** 1 Shenzhen Eye Hospital, Shenzhen Eye Medical Center, Southern Medical University, Shenzhen, China; 2 Shenzhen International Graduate School, Tsinghua University, Shenzhen, China; 3 Nanshan College, Guangzhou Medical University, Guangzhou, China

**Keywords:** granular corneal dystrophy type 2, LASIK, TGFBI mutation, lamellar keratoplasty, refractive surgery exacerbation

## Abstract

**Purpose:**

To demonstrate that LASIK is contraindicated in patients with granular corneal dystrophy through documentation of post-surgical disease exacerbation.

**Methods:**

Comprehensive clinical evaluation including anterior segment photography and multimodal imaging were performed on the proband and affected family members. Genomic DNA was isolated from peripheral blood samples, and targeted sequence capture array technique followed by PCR and Sanger sequencing were employed for mutation identification.

**Results:**

The proband presented with bilateral progressive vision loss following LASIK surgery performed at age 18. Anterior segment photography revealed extensive granular opacification within the corneal stroma, predominantly at the LASIK flap interface. Genetic analysis identified a heterozygous c.371G > A mutation in exon 4 of the *TGFBI* gene, resulting in a p. R124H substitution. This identical mutation was confirmed in 14 of 37 tested family members, demonstrating autosomal dominant inheritance.

**Conclusion:**

LASIK surgery significantly accelerates granular corneal dystrophy type 2 progression through multiple pathophysiological mechanisms. Comprehensive preoperative assessment incorporating genetic screening for *TGFBI* mutations is essential to identify and exclude contraindicated candidates for refractive surgery.

## Introduction

Corneal dystrophies represent a heterogeneous group of rare, inherited disorders characterized by progressive accumulation of abnormal deposits within specific corneal layers, ultimately compromising visual function (1). The 2015 International Classification of Corneal Dystrophies categorizes these conditions into four distinct groups: epithelial and subepithelial dystrophies, epithelial-stromal *TGFBI* dystrophies, stromal dystrophies, and endothelial dystrophies (2, 3). Among these, granular corneal dystrophy (GCD) stands as a paradigmatic example of an autosomal dominant stromal dystrophy, arising from mutations in the *TGFBI* gene located on chromosome 5q31 (4). This genetic locus harbors mutations responsible for several hereditary corneal dystrophies, including Reis-Bücklers dystrophy, Thiel-Behnke dystrophy, lattice corneal dystrophy, and both subtypes of granular corneal dystrophy (5).

Granular corneal dystrophy type 2 (GCD2), initially described by Bücklers in 1938 and formerly known as Avellino corneal dystrophy, was historically considered a milder variant of GCD1 for nearly a century (2). This condition exhibits a strong genotype–phenotype correlation with the R124H mutation in the TGFBI gene, which was first identified in families from the Avellino region of Italy (2, 6, 7). Distinguished from GCD1 by its later onset, slower progression, and milder visual impairment, GCD2 nonetheless presents significant clinical challenges, particularly in homozygous individuals who experience accelerated disease progression and earlier symptom onset (8, 9). Epidemiological studies reveal a notably higher prevalence of GCD2 in East Asian populations compared to European cohorts, suggesting potential ethnic variations in genetic susceptibility or environmental modifying factors (10, 11).

The pathophysiology of GCD2 involves complex interactions between genetic predisposition and environmental triggers (8). Mechanical corneal injury has been established as a potent inducer of *TGFBI* overexpression, initiating cascades of extracellular matrix remodeling that accelerate the deposition of TGF-β-induced protein aggregates within the corneal stroma (12). This mechanistic understanding has particular relevance for refractive surgery, where multiple reports have documented dramatic exacerbation of corneal deposits following various laser procedures, transforming subclinical disease into visually significant pathology (13–16). Here, we present a comprehensive analysis of bilateral GCD2 exacerbation following LASIK surgery, including detailed clinical, genetic, and histopathological findings, with successful visual rehabilitation through lamellar keratoplasty.

## Case presentation

### Initial presentation and clinical history

A 25-year-old male presented to our cornea service with progressive bilateral visual deterioration over the preceding four years. His ocular history was significant for bilateral LASIK surgery performed at age 18 for myopia correction at an external facility. The patient reported satisfactory initial postoperative vision that gradually deteriorated beginning approximately three years after the procedure, with acceleration of symptoms over the past year. He reported no history of ocular trauma, infection, or inflammation following the refractive surgery. His medical history was unremarkable, with no systemic conditions or regular medications. The patient reported no known allergies and denied smoking or alcohol use. Family history revealed that both his father and sister had been diagnosed with “corneal spots” but maintained functional vision without requiring intervention. No consanguinity was reported in the family. The patient expressed concern about his rapidly progressing visual impairment, which was significantly impacting his professional activities as an office worker requiring extensive computer use.

### Clinical and imaging evaluation

**Figure 1:**
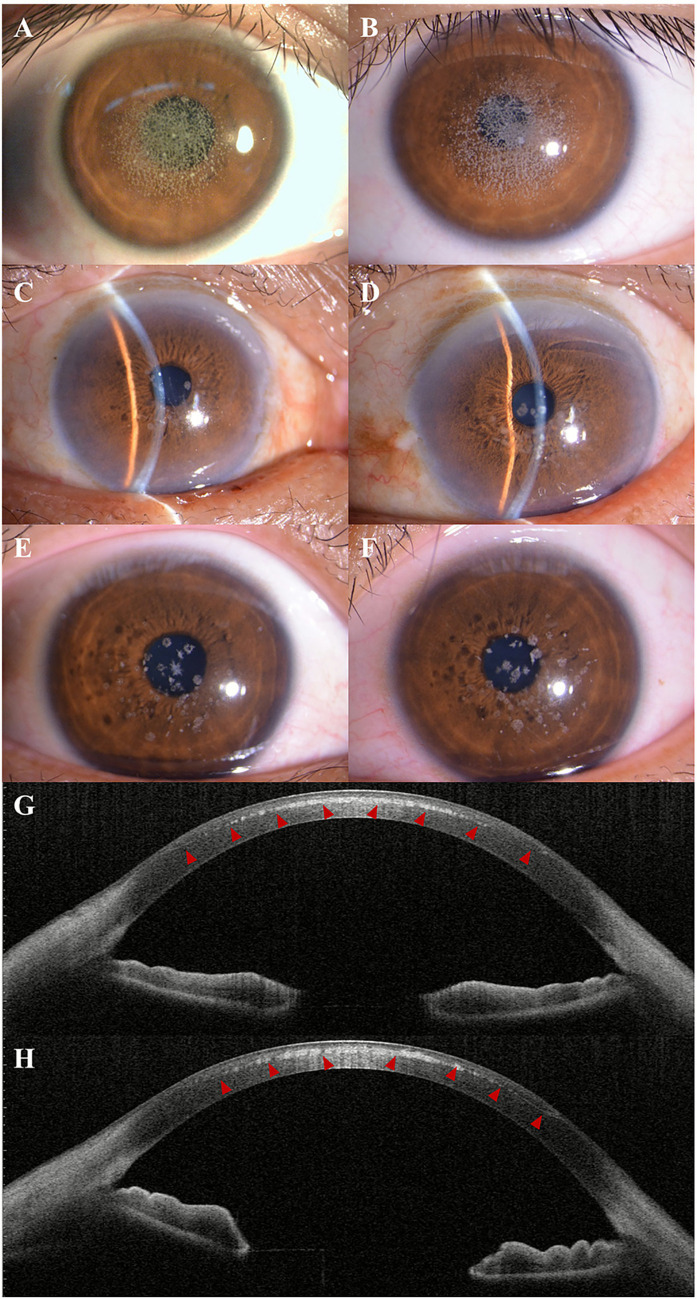
Clinical and imaging manifestations of granular corneal dystrophy type 2 in the proband and affected family members. Slit-lamp biomicroscopy photographs demonstrating extensive coalescent white-gray granular opacities throughout the central and paracentral corneal stroma in the proband’s right eye **(A)** and left eye **(B)**, seven years after LASIK surgery. The deposits exhibit a characteristic “crumb-like” appearance with irregular borders and variable density. Comparative examination of the proband’s father reveals bilateral granular deposits predominantly in the superficial and mid-stroma (**C**, right eye; **D**, left eye) without prior surgical intervention. The proband’s sister shows mild bilateral granular opacities (**E**, right eye; **F**, left eye) with preserved visual function. Anterior segment optical coherence tomography demonstrates hyperreflective deposits concentrated at the LASIK flap interface, creating an irregular band at approximately one-third to one-half corneal depth in both eyes (**G**, right eye; **H**, left eye), with relative sparing of the posterior stroma.

Comprehensive ophthalmological examination of the proband revealed best-corrected visual acuity (BCVA) of 0.1 (20/200) in the right eye and 0.2 (20/100) in the left eye, representing a marked decline from his reported post-LASIK acuity. Intraocular pressure measured 16 mmHg in the right eye and 15 mmHg in the left eye by Goldmann applanation tonometry. Pupillary responses were normal bilaterally with no relative afferent pupillary defect. Slit-lamp biomicroscopy demonstrated striking bilateral corneal changes characterized by extensive coalescent granular opacities throughout the central and paracentral corneal stroma, predominantly at the LASIK flap interface, with a “crumb-like” appearance typical of GCD2 (Figure 1A, right eye; Figure 1B, left eye). The deposits appeared as discrete white-gray granules that coalesced into larger, irregular opacities. The corneal epithelium appeared intact without evidence of erosions or irregularities. The anterior chamber was deep and quiet bilaterally, and the crystalline lenses were clear. Screening of family members revealed milder corneal changes. The patient’s 52-year-old father demonstrated bilateral scattered white stromal deposits in a characteristic granular pattern, though less extensive than those observed in the proband, with preserved visual acuity of 20/25 in both eyes (Figure 1C, right eye; Figure 1D, left eye). Similarly, the patient’s 27-year-old sister exhibited bilateral granular opacities of mild severity, maintaining visual acuity of 20/20 bilaterally (Figure 1E, right eye; Figure 1F, left eye). Neither family member had undergone ocular surgery, and both were asymptomatic regarding their vision. Anterior segment optical coherence tomography (AS-OCT) revealed hyperreflective deposits concentrated at the LASIK flap interface, located at approximately one-third to one-half of the total corneal thickness, creating an irregular, discontinuous hyperreflective band (Figure 1G, right eye; Figure 1H, left eye). The posterior stroma appeared relatively unaffected, maintaining normal reflectivity patterns.

### Genetic analysis and family pedigree

**Figure 2:**
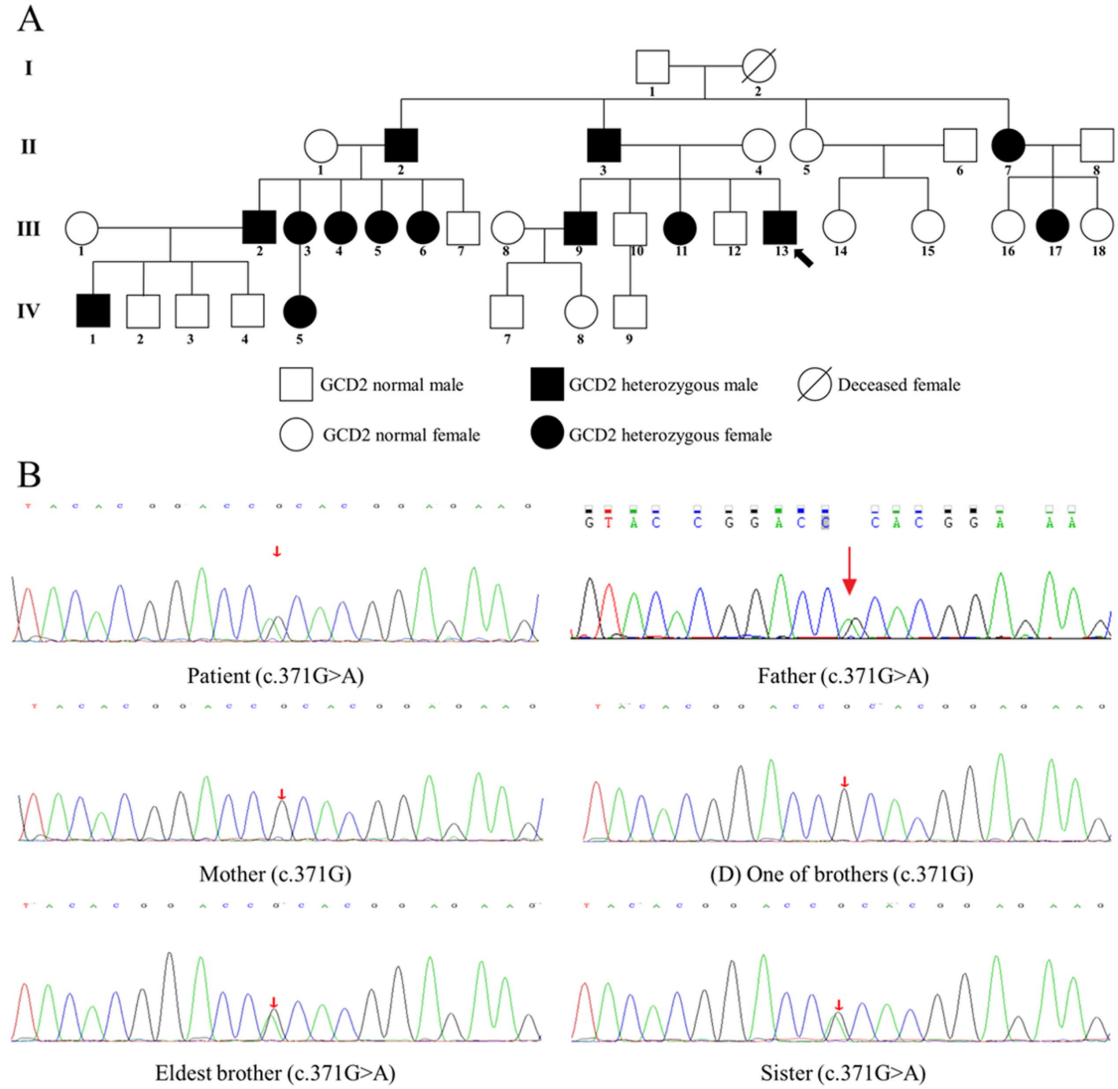
Genetic analysis and family pedigree of autosomal dominant GCD2. **(A)** Three-generation pedigree. Squares represent males; circles represent females. Filled symbols indicate clinically affected individuals with confirmed mutations. Half-filled symbols denote mutation carriers with a subclinical phenotype. The arrow indicates the proband (III-13). The inheritance pattern demonstrates male-to-male transmission. **(B)** Direct sequencing chromatograms of TGFBI exon 4. The heterozygous c.371G > A transition (arrows) results in the p. R124H substitution in the affected proband (III-13), his affected father (II-3), and his affected sister (III-11). The wild-type sequence (G/G) is shown in the unaffected mother (II-4). Both wild-type (G) and mutant **(A)** peaks are visible in the heterozygous individuals.

Following approval from the Ethics Committee of Shenzhen Eye Hospital and obtaining informed consent from all participants, we performed comprehensive genetic analysis on the proband and 37 family members across three generations. Genomic DNA extracted from peripheral blood underwent targeted sequence capture array analysis screening 281 genes associated with corneal diseases. Genetic sequencing revealed a heterozygous c.371G > A mutation in exon 4 of the TGFBI gene in the proband (III-13), resulting in an arginine to histidine substitution at codon 124 (p. R124H). This pathogenic variant was confirmed through PCR amplification and Sanger sequencing. Segregation analysis within the family demonstrated perfect co-segregation of the mutation with the clinical phenotype (Figures 2A,B). The proband’s father (II-3), eldest brother (III-9), and sister (III-11) all carried the identical heterozygous c.371G > A mutation. Conversely, the unaffected mother (II-4) and one brother (III-12) showed wild-type sequences at this locus, confirming the autosomal dominant inheritance pattern characteristic of GCD2.

### *In vivo* confocal microscopy

**Figure 3:**
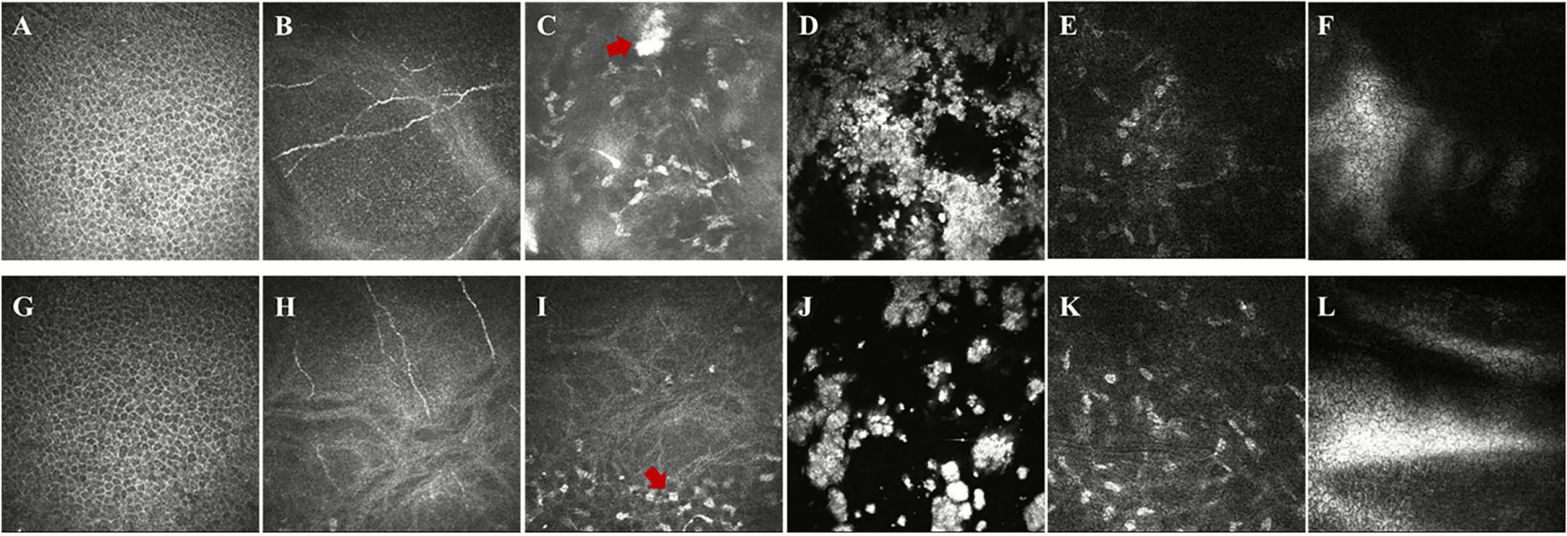
*In vivo* confocal microscopy revealing layer-specific corneal alterations following LASIK in granular corneal dystrophy type 2. High-resolution confocal images from the right eye **(A–F)** and left eye **(G–L)** at various corneal depths. The superficial epithelium maintains normal honeycomb architecture with regular polygonal cells **(A,G)**. Subbasal nerve plexus between the basal epithelium and Bowman’s membrane shows preserved dendritic branching patterns **(B,H)**. Anterior stroma reveals multiple hyperreflective granular deposits of varying sizes disrupting normal architecture **(C,I)**. Mid-stromal level, corresponding to the LASIK interface, shows coalescent dense aggregates creating significant light scatter **(D,J)**. Deep stroma demonstrates minimal involvement with preserved keratocyte morphology **(E,K)**. Endothelial mosaic displays regular hexagonal morphology with maintained cell density **(F,L)**. Field of view: 400 × 400 μm.

*In vivo* confocal microscopy (IVCM) (17) offered cellular-level visualization of the corneal alterations. The epithelial layer maintained its normal honeycomb architecture with regular cellular morphology and tight intercellular junctions. Subbasal nerve fibers demonstrated preserved dendritic branching patterns without evidence of neurotrophic changes. The anterior and mid-stromal layers revealed the pathognomonic findings of GCD2, with numerous hyperreflective granular deposits of varying sizes and morphologies scattered throughout these layers. These deposits appeared as discrete, highly reflective structures disrupting the normal stromal architecture. In contrast, the deep stromal layer showed minimal involvement, with keratocytes maintaining their characteristic stellate appearance. The endothelial mosaic displayed regular hexagonal morphology with uniform cell size and preserved cell density of approximately 2,500 cells/mm^2^, indicating absence of endothelial dysfunction (Figures 3A–L).

### Surgical intervention and histopathological findings

**Figure 4:**
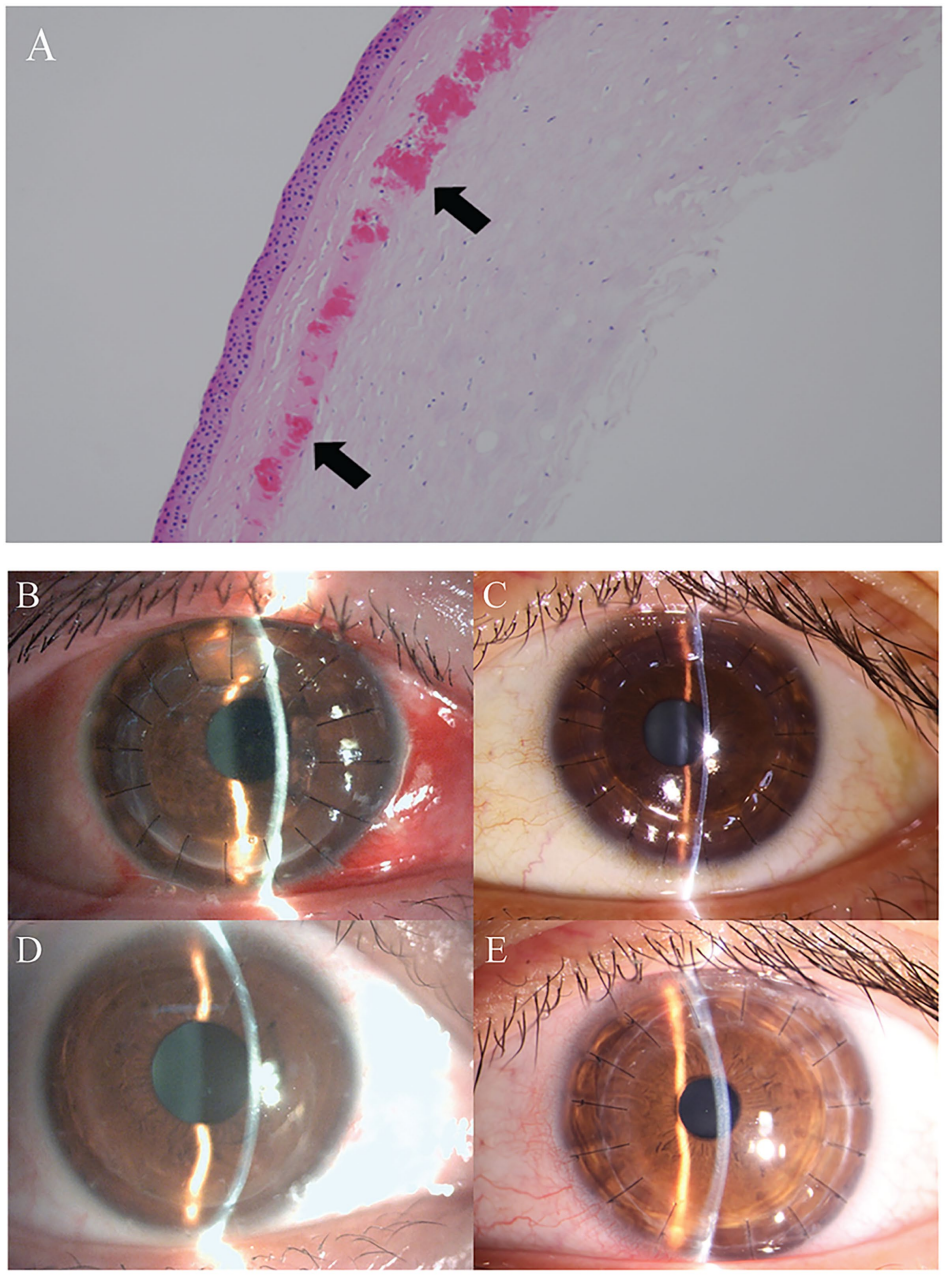
Histopathology and postoperative outcomes. **(A)** Hematoxylin and eosin staining of the excised corneal tissue showing multiple eosinophilic granular deposits (arrows) concentrated at the presumed LASIK interface, disrupting the normal collagen lamellar arrangement. Original magnification ×100. Slit-lamp photographs showing clear corneal grafts at postoperative day 3 in right **(B)** and left **(C)** eyes with excellent graft-host apposition and radially placed 10–0 nylon sutures. Long-term follow-up demonstrates sustained graft clarity without disease recurrence: right eye at 5 years **(D)** with selective suture removal and excellent visual rehabilitation; left eye at 6 months **(E)** showing maintained transparency with ongoing visual improvement. Arrows in **(D)** indicate well-healed graft-host junction.

Given the severity of visual impairment and the extensive corneal opacification, the patient underwent sequential bilateral deep anterior lamellar keratoplasty. The right eye surgery was performed in April 2020 under general anesthesia, followed by the left eye procedure in February 2025. The surgical technique involved creation of an 8.0-mm diameter trephination to approximately two-thirds corneal depth using a vacuum trephine system. Manual lamellar dissection was then carefully performed using specialized corneal dissection instruments, progressing to the deep stromal layers while preserving Descemet’s membrane and endothelium. The dissection revealed densely opacified tissue at the LASIK interface, requiring meticulous separation. The residual stromal bed appeared relatively clear, supporting the decision for lamellar rather than penetrating keratoplasty. The donor corneal tissue, prepared with removal of Descemet’s membrane and endothelium, was trephined to 8.5 mm to ensure optimal graft-host apposition. The graft was secured with sixteen interrupted 10–0 nylon sutures placed in a radial configuration to minimize astigmatism. Intraoperative examination confirmed watertight wound closure and appropriate anterior chamber depth. Histopathological examination of the excised corneal tissue demonstrated characteristic features of GCD2. Hematoxylin and eosin staining revealed multiple eosinophilic granular deposits scattered throughout the superficial and mid-stromal layers, with the highest concentration at the presumed LASIK interface level. Special stains including Masson’s trichrome confirmed the proteinaceous nature of the deposits (Figure 4A).

### Postoperative course and outcomes

The postoperative regimen consisted of levofloxacin 0.5% eye drops four times daily for two weeks, combined tobramycin-dexamethasone drops four times daily for four weeks, followed by a gradual taper. Tobramycin-dexamethasone ointment was applied nightly for four weeks. Long-term immunosuppression was maintained with tacrolimus 0.03% eye drops twice daily, along with preservative-free artificial tears for ocular surface support. Early postoperative examinations revealed clear corneal grafts with excellent graft-host apposition and no evidence of interface haze or opacity (Figures 4B–E). The anterior chambers remained deep and quiet without signs of inflammation or rejection. Serial examinations demonstrated progressive visual rehabilitation, with the right eye achieving BCVA of 20/20 with rigid gas-permeable contact lens correction at the five-year follow-up visit. The left eye, six months postoperative at the time of this report, has achieved BCVA of 20/50 with ongoing improvement anticipated. Throughout the follow-up period, both grafts have maintained excellent clarity without evidence of GCD2 recurrence at the graft-host interface or within the donor tissue. Regular endothelial cell counts have shown stable cell density, and corneal topography has demonstrated regular astigmatism amenable to contact lens correction. The patient has returned to full professional activities and reports high satisfaction with the surgical outcomes.

## Discussion

This case exemplifies the critical importance of recognizing *TGFBI*-related corneal dystrophies as absolute contraindications to laser refractive surgery. The dramatic exacerbation of GCD2 following LASIK in our patient, transforming from subclinical disease to severe visual disability within seven years, underscores the complex pathophysiological interactions between surgical trauma and genetic predisposition to abnormal protein deposition.

The *TGFBI* gene, located on chromosome 5q31 and comprising 17 exons, encodes a 683-amino acid protein known as kerato-epithelin. This protein serves essential roles in maintaining corneal transparency through regulation of cell adhesion, proliferation, and differentiation via interactions with extracellular matrix components including laminin, fibronectin, and various collagens (18). The R124H mutation identified in our patient and affected family members represents the most common pathogenic variant associated with GCD2, causing structural alterations in kerato-epithelin that promote abnormal protein aggregation and deposition within the corneal stroma (19).

The mechanisms underlying post-LASIK exacerbation of GCD2 are multifactorial and synergistic. Surgical trauma induces immediate upregulation of *TGFBI* gene expression, accelerating the production and subsequent aggregation of mutant kerato-epithelin (20, 21). The inflammatory cascade triggered by laser ablation releases cytokines including interleukin-6, transforming growth factor-alpha, and tumor necrosis factor-alpha, which further promote protein deposition and corneal opacification (16, 22, 23). The creation of a potential space at the flap-stromal interface provides an anatomical niche for preferential protein accumulation, extending beyond the typical anterior stromal distribution of GCD2 deposits (14, 24).

Additionally, the biomechanical alterations induced by stromal ablation may compromise the structural integrity of the cornea, potentially triggering keratocyte apoptosis and accelerating dystrophic progression (25, 26). The disruption of normal stromal architecture may impair the clearance mechanisms for abnormal protein deposits, creating a self-perpetuating cycle of accumulation (27). Furthermore, potential complications such as interface fluid accumulation or epithelial ingrowth, as observed in some post-LASIK GCD2 cases, can further compromise visual outcomes (28, 29).

The successful visual rehabilitation achieved through lamellar keratoplasty in our patient demonstrates the efficacy of this approach for managing severe post-LASIK GCD2 exacerbation. The decision to perform deep anterior lamellar keratoplasty rather than penetrating keratoplasty was based on the preserved endothelial function and the predominantly anterior stromal localization of deposits. This technique offers the advantages of eliminating endothelial rejection risk while maintaining the structural integrity of the globe. The absence of recurrence at five years in the right eye and six months in the left eye is encouraging, though continued surveillance remains essential given reports of late recurrence in some GCD2 patients following keratoplasty (24, 30).

Our findings reinforce the critical importance of comprehensive preoperative evaluation for all refractive surgery candidates. The presence of even subtle corneal opacities, particularly in individuals of East Asian descent where GCD2 prevalence is higher, should prompt careful slit-lamp examination and consideration of genetic testing. Family history of corneal disease, even if described vaguely as “corneal spots” or “cloudiness,” warrants thorough investigation. The implementation of routine *TGFBI* genetic screening for refractive surgery candidates in endemic populations has been advocated by several authors and may prevent devastating outcomes like those observed in our patient (10, 11).

In conclusion, this case provides compelling evidence that LASIK and other forms of laser refractive surgery are absolutely contraindicated in patients with GCD2, even when the disease is subclinical or undiagnosed. The potential for severe, progressive visual loss following surgery mandates meticulous preoperative screening combining detailed clinical examination with genetic testing when indicated. As the prevalence of both refractive surgery and recognition of *TGFBI*-related dystrophies continues to increase, establishing standardized screening protocols becomes increasingly crucial for preventing these preventable complications. Future research should focus on developing rapid, cost-effective screening methods for *TGFBI* mutations and investigating potential therapeutic interventions to prevent or reverse abnormal protein deposition in affected individuals.

## Data Availability

The original contributions presented in the study are included in the article/supplementary material, further inquiries can be directed to the corresponding author/s.
